# The Gut Microbiota Impacts Gastrointestinal Cancers through Obesity, Diabetes, and Chronic Inflammation

**DOI:** 10.3390/life14101219

**Published:** 2024-09-24

**Authors:** Konstantin A. Rumyantsev, Vera V. Polyakova, Irina V. Sorokina, Polina S. Feoktistova, Igor E. Khatkov, Natalia A. Bodunova, Lyudmila G. Zhukova

**Affiliations:** Loginov Moscow Clinical Scientific Center, 111123 Moscow, Russia

**Keywords:** gut microbiota, obesity, diabetes, chronic inflammation

## Abstract

The gut microbiota’s pivotal role in human health is increasingly evident, particularly in chronic conditions like obesity, diabetes, and inflammatory diseases. This intricate symbiotic relationship influences metabolic balance and immune responses. Notably, gut microbial dysbiosis is linked to obesity’s metabolic disruption and chronic low-grade inflammation. Similarly, in diabetes, the microbiota’s impact on insulin resistance and glucose metabolism is becoming evident. Chronic inflammation, a common denominator in these conditions, is also a recognized precursor to carcinogenesis. This intersection prompts a compelling question: does the gut microbiota’s influence extend to gastrointestinal cancers like colorectal and pancreatic cancer? These malignancies are closely intertwined with inflammation and metabolic dysregulation. Exploring whether the microbial signatures associated with chronic conditions overlap with precancerous or cancerous states offers new perspectives. This article reviews emerging evidence on the interplay between the gut microbiota, chronic conditions, and gastrointestinal cancers. By elucidating these connections, we aim to uncover potential avenues for innovative diagnostic, preventative, and therapeutic strategies in colorectal and pancreatic cancer management.

## 1. Introduction

The human microbiome is a set of microbes, their genes, and their products that colonize the body from birth and are usually transferred to a child from its mother. The microbiota of each person is unique and differs both at the species and strain levels. The gut microbiota is an ecosystem consisting of a community of microorganisms living in our gastrointestinal tract (GI) from the oral cavity to the colon [1]. The role of the gut microbiota in diagnosing and monitoring the progress of different human diseases and health conditions has grown thanks to the broader use and decreasing cost of deep-sequencing technologies [2]. The new approach to studying microbial communities has paved the way to new analytical depths, revealing connections between not only diseases of the GI but also cancers and systemic conditions.

**Figure 1:**
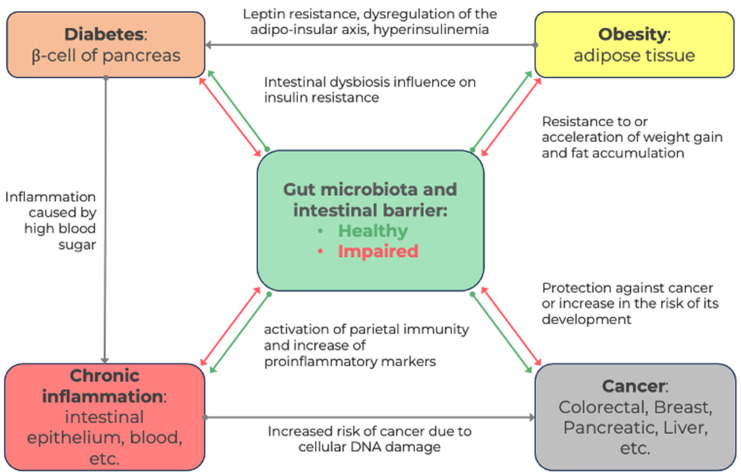
A diagram of the relationships between the intestinal microbiota and various pathological conditions, describing the complex nature of signals and the clockwise path from obesity to cancer through diabetes and chronic inflammation.

Currently, changes in the composition of the intestinal microbiota are associated with many morbid conditions, including diabetes mellitus and inflammatory bowel diseases. Growing evidence has indicated that the intestinal microbiota have key roles in the carcinogenesis of many tumor types [3]. It has been assumed that various molecular pathways of pathogenesis are affected, explaining how intestinal bacteria can be either protective against a disease or promote it (Figure 1). [4]. 

The participation of the intestinal microbiota in forming immune responses in healthy people of different ages is one of the leading mechanisms of this influence on the body. The accumulating data have focused on the importance of using probiotics and microbiota transplants in the treatment of immune-mediated disorders, metabolic syndrome, and cancer.

The study of the intestinal microbiota, in addition to its fundamental importance, is relevant in the clinical aspect. There is growing evidence that the gut microbiome is most actively involved in the pathogenesis of malignant neoplasms of the digestive system due to changes in the quantitative and qualitative compositions of bacteria. Data on the relationship between the composition and metabolic characteristics of the microbiome in various tumor types have opened up prospects for its use in diagnosis, treatment, and cancer prevention.

The gut microbiota, often regarded as a silent orchestrator of health and disease, has emerged as a pivotal player in the multistage process of carcinogenesis. One of the intriguing mechanisms through which gut microbiota contribute to this complex journey is by increasing the production of genotoxic bacterial metabolites [5]. Within the intricate landscape of the gastrointestinal tract, certain bacterial species can generate metabolites with genotoxic potential, such as reactive oxygen species, secondary bile acids, and biogenic amines. These metabolites can directly damage the DNA of host cells, initiating genomic instability and mutations—one of the fundamental hallmarks of cancer development. As the gut microbiota composition shifts, particularly in conditions like dysbiosis and chronic inflammation, the balance between beneficial and detrimental microbial metabolites can tip towards the latter, amplifying the risk of carcinogenesis. This revelation underscores the importance of understanding the complex interplay between the gut microbiota and the intricate molecular events driving cancer, offering a promising avenue for potential interventions and therapeutic strategies in cancer prevention and management.

The probable mechanisms of microorganism participation in the development of colorectal cancer, pancreatic cancer, and diabetes mellitus are discussed below.

In this review, we briefly describe methods for microbiota composition analysis, mention existing bottlenecks regarding the use of microbiota in clinical trials, and discuss other concerns regarding the use of the microbiota in clinical practice and the development of treatment algorithms that include analyses of gut health via studying microbiota composition. We aim to emphasize the need to look at a broader picture that includes health conditions that could impact cancer indirectly through the gut microbiota, including obesity, diabetes, and chronic inflammation. Finally, we also consider the potential influence of the composition of the intestinal microbiota on cancer immunotherapy, a connection that has been uncovered only recently.

## 2. Evolution of Approaches to the Analysis of Intestinal Microbiota

Presently, there are many different approaches to identifying and diagnosing the human intestinal microbiota, including biochemical and molecular genetic methods, each with its advantages and limitations [6].

Bacteria cultivation on various selective media is one of the first and simplest methods to study the intestinal microbiota. This technique involves the use of selective nutrient media that allow the growth of a limited set of microorganisms. Thus, colonies grown on a selective medium make it possible to qualitatively confirm their presence in an individual’s gut. Moreover, the study of new multi-compound and rich culture media has made it possible to cultivate and distinguish up to 500 species of microorganisms, which is several times higher than is available with conventional analysis [7]. However, this method does not allow a complete assessment of the entire human microbiota, nor can it detect some intraspecies differences in the bacteria. Although this approach has not been changed for a long time, selective media cultivation remains widespread [8].

The development of molecular genetic methods has contributed to the introduction of such methods as the quantitative polymerase chain reaction (qPCR) into clinical practice. The qPCR measures the abundance of specific microbial taxa by amplifying and quantifying target genes specific to a particular species or genus of microorganisms. qPCR is a rapid and relatively low-cost technique, making it suitable for the routine analysis of known pathogens. However, as with selective media, qPCR diagnostics are only capable of identifying a limited range of bacterial species.

The most influential changes in approaches to studying microbiota can be attributed to the advent of 16S rRNA sequencing, which has made it possible to read hypervariable regions in the DNA of bacteria and, thereby, distinguish new species and classify them. In more detail, this method targets the conserved region of the 16S ribosomal RNA gene, which is present in the genomes of almost all bacteria and archaea. PCR amplification of this gene is performed using universal primers, followed by high-throughput sequencing. The resulting sequences can be clustered into operational taxonomic units (OTUs) to determine the diversity and relative abundance of different microbial taxa. Thanks to this technique, for the first time, it was possible to identify up to 75% of new bacterial species that had not previously been isolated as a pure culture, which made 16S rRNA sequencing the most promising method for gut microbiota analysis [9,10,11,12].

However, the 16S rRNA sequencing approach also has limitations in terms of the depth of sequencing and shortness of the studied regions that limit the potential diversity of the region and, hence, the number of reliably detected bacterial species. In this regard, many researchers are switching to shotgun metagenomics. In this approach, the entire DNA content of a microbial sample is sequenced. It provides a more comprehensive view of the microbial community as it can identify bacteria and other microorganisms like viruses and fungi. Shotgun metagenomics allows for the reconstruction of entire microbial genomes, enabling a more in-depth functional analysis [13].

Several analytical approaches are currently used less frequently than others, including quantitative polymerase chain reaction (qPCR) and metabolomics. The qPCR method measures the abundance of specific microbial taxa by amplifying and quantifying target genes specific to a particular species or genus of microorganisms. qPCR is a rapid and relatively low-cost technique, making it suitable for the routine analysis of known pathogens. However, this approach rarely succeeds in discovering meaningful data in exploratory research. Metabolomics, while not directly analyzing microbial DNA, studies the metabolites produced by a microbial community. Metabolites can provide valuable information about the functional activities of the microorganisms present and the interactions within the community. This analytical approach is regaining its importance due to the growing volume of data and analytical capabilities, which have been primarily related to the development of computational analysis [14,15]. Metabolomics is not only becoming relevant but also showing strong and promising results that will lead to radical changes in medicine.

When conducting microbiota composition analyses, researchers often use a combination of these methods to comprehensively understand a microbial community’s structure, diversity, and functional potential. Each method has its strengths and weaknesses, and the choice of the technique depends on the specific research question and available resources. Additionally, data analysis and bioinformatics play crucial roles in making sense of the vast amount of sequencing data generated by these methods.

## 3. Gut Microbiota in the Development of Obesity: Protection and Promotion

Obesity is the initial pathophysiological mechanism of many diseases, for example, DM2, hypertension, and cancer (Figure 1). The pathogenesis of obesity is multifactorial, but one of the most interesting factors studied recently is the influence of the gut microbiota.

The composition, diversity index, relative levels, and functional pathways of the microbiome may predispose an adult to obesity [16]. The gut microbiota has been linked to the control of food intake and satiety by intestinal peptide signaling, where bacterial products activate enteroendocrine cells by modulating the paracrine signaling molecules produced by the enterocyte [17]. The gut microbiota can increase the production of certain short-chain bile acids associated with the increased production of peptide, ghrelin, insulin, and glucagon-like peptide-1 [18].

It has been suggested that certain groups of bacteria effectively absorb nutrients, and due to the rapid metabolism of these nutrients, they increase the amount of calories absorbed, which leads to an increase in body mass index (BMI) [19].

The number of bacteria belonging to the Bacteroidetes type affects glucose intolerance caused by the consumption of high-fat foods [20]. Another putative pathway of the microbiota contributing to obesity is anorexigenic intestinal GLP-1, which may be a promising strategy for treating obesity. The intestinal microbiota participates in the metabolism of bile acids through the processes of deconjugation and dehydroxylation in the intestinal lumen. They turn primary BAs into secondary BAs, as follows: cholate into deoxycholate and henodeoxycholate into litocholate [21]. This action is due to the enzymes in bile salt hydrolase, which are present mainly in *Firmicutes* and *Bacteroidetes* and especially in clusters of the genera *Clostridium* [22]. When the enzymatic action of the microbiota changes, the same thing happens with the composition of the BAs, facilitating the absorption of fat and causing obesity [23]. Intestinal dysbiosis can alter the production of the gastrointestinal peptides associated with saturation, which leads to an increase in food intake. In obese people, this dysbiosis appears to be associated with increases in *Firmicutes*, the genus *Clostridium*, and the species *Eubacterium rectale*, *Clostridium coccoides*, *Lactobacillus reuteri*, *Clostridium histolyticum*, *Staphylococcus aureus*, and *Akkermansia muciniphila* [24]. A systematic review of 12,500 studies also identified an association between obesity and the *Firmicutes/Bacteroidetes* ratio, among the overabundance of other genera like *Fusobacteria*, *Proteobacteria*, *Mollicutes*, and *Lactobacillus* [25].

The recently described *Christensenellaceae* family has been associated with reduced adipose tissue formation both in human correlation analyses and in animal experiments. It is important to note that *Christensenellaceae* and *C. minuta,* in particular, were also associated with certain genetic characteristics of their host, e.g., with the interleukin 23 receptor (ILR23) and fucosyltransferase 2 (FUT2) genes [26].

A significant relationship was shown to exist between changes in the microbiota and the clinical markers of patients who underwent bariatric surgery (a bypass gastric anastomosis, according to Ru). An increase in bilirubin is associated with increases in the taxa *Prevotellaceae*, *Bacteroidales*, and *Peptococceae*; an increase in iron content is associated with an increase in the number of *Pasteurellaceae*; and a decrease in HbA1c is associated with a decrease in *Coriobacteriacea* and an increase in *Clostridiales* taxa. The most pronounced positive association has been described between *Lachnospiraceae* and *Coriobacteriaceae* taxa in lowering cholesterol levels [27].

## 4. Mutual Influence of Diabetes on Gut Microbiota and Gut Microbiota on Diabetes

The gut microbiota plays an important role in the development of metabolic diseases, especially type 2 diabetes (DM2). *Acidaminococcales*, *Bacteroides plebeius*, and *Phascolarctobacterium* may be potential biomarkers for DM2.

Changes in the gut microbiota may also be associated with insulin and glucose resistance. The role of the intestinal microbiota in the regulation of glucose and insulin sensitivity has been noted in the literature. There is an assumption that there is a connection between the bioavailability, bioactivity, and effects of some biologically active substances entering the body with food and the intestinal microbiota, starting with bacterial conversion [28].

The gut microbiota is a modifiable determinant of DM2, and it is closely related to dietary factors. It is assumed that the pathological effect of intestinal microbiota dysbiosis on carbohydrate metabolism consists of increased intestinal permeability, the increased absorption of lipopolysaccharides, the synthesis of short-chain bile acids, the pathologically altered conversion of primary bile acids into secondary bile acids, and the increased bacterial production of toxic substances such as trimethylamine N-oxide. This leads to the activation of inflammatory processes and autoimmune pathways and, subsequently, to the disruption of insulin signaling. The course of DM2 can be improved by modifying the intestinal microbiota, which helps restore impaired glucose tolerance. The study of the molecular mechanisms linking a host and its intestinal microbiota in DM2 may provide new ideas for the treatment of DM2 [29]. Other findings have indicated that gut dysbiosis associated with DM2 is similar to changes associated with obesity [30,31]. It is important to note that in these studies, DM2 was also found to be associated with several pro-inflammatory bacteria (e.g., *Roseburia*, *Lachnospira*, *Coprococcus*, *Phascolarctobacterium*, *Blautia*, and *Anaerostipes*), which indicates a possible further path of the disease to chronic inflammation and cancer.

## 5. Increased Risk of Colorectal Cancer through Microbiota-Induced Inflammation

Colorectal cancer (CRC) ranks third in the incidence structure, accounting for 1,800,000 new cases of cancer in 2018 [32]. The development of CRC is associated with several risk factors such as age greater than 50 years old, a personal or family history of adenomatous polyps, inherited syndromes, smoking, alcohol use, and, of special interest, obesity, DM2, and a personal history of inflammatory bowel diseases, all of which are proven factors of the gut microbiota composition’s clinical impacts. In CRC, metabolites of the intestinal microbiome have tumor-like or antitumor characteristics. Lipopolysaccharides expressed in colonocytes inhibit cell death, activate cellular immune response via TLR2, and stimulate signal transmission to produce pro-inflammatory cytokines that trigger carcinogenesis. Lipoteichoic acid is a part of the cell wall of a Gram-positive bacterium and is considered an analog of lipopolysaccharide, a component of the cell wall of a Gram-negative bacterium. Eating foods with a high-fat content increases the relative number of sulfate-reducing bacteria, such as *Desulfovibrio vulgaris*, which is involved in the metabolism of bile acids, for example, and lithocholic and deoxycholic, which are known for their potential tumor activity. Butyric acid is an important short-chain fatty acid that is formed from fermentable fibers when feeding intestinal bacteria, and it has been shown to have antitumor properties.

Chronic inflammation is mediated by inflammatory mediators (for example, tumor necrosis factor (TNF) and cytokines such as IL 6, 1β, etc.) that activate transcription nuclear factor (NF-kB), promoting carcinogenesis in the colon [33].

The risk of CRC development is correlated with microorganisms such as *Streptococcus bovis*, enterotoxigenic *Bacteroides fragilis*, *Fusobacterium nucleatum*, *Enterococcus faecalis*, *Escherichia coli,* and *Peptostreptococcus anaerobius* [34]. The presence of the *F. nucleatum* microorganism is associated with poor prognoses in patients with CRC. According to the results of intestinal microbiota sequencing, it was shown that in a patient with CRC, there is a less diverse microbiota composition, and the number of *Basidiomycota*: *Ascomycota* is increased compared to a healthy person. The characteristics of intestinal microbiomes also vary geographically, but many common bacterial strains associated with the development of CRC have been found in different populations around the world. Among them, the following seven enriched bacterial strains associated with CRC have been identified: *Bacteroides fragilis*, *Fusobacterium nucleatum*, *Parvimonas micra*, *Porphyromonas asaccharolytica, Prevotella intermedia*, and *Thermanaerovibrio acidaminovorans* [35].

Comparatively, the gut microbiota of an individual with CRC has a different composition of bacterial strains than a healthy gut microbiome and includes strains individually associated with CRC, such as *Bacteroides fragilis*, *Streptococcus gallolyticus*, *Enterococcus faecalis*, and *Escherichia coli*. Higher amounts of these bacterial strains in the fecal and tumor samples of patients with tumor microbiota CRC may serve as biomarkers of CRC. The intestinal microbiota could be involved in the initiation, progression, and metastasis of CRC. A change in the composition of the intestinal microbiota can serve as a diagnostic sign and confirm the presence of CRC in the early stages of the disease, and this requires a possibly easier and cheaper determination method than the ctDNA currently accepted according to the recognized clinical guidelines [36].

## 6. Limited Evidence of Gut Microbiota Influence on the Incidence of Pancreatic Cancer

Pancreatic ductal adenocarcinoma (PDAC) represents one of the most aggressive solid tumors with a dismal prognosis, increasing incidence, and limited therapeutic options. It is the fourth most common cause of cancer death worldwide, and it is projected to become the second most common cause of cancer death by 2030 [37]. While overall cancer survival has shown moderate improvement with complex treatment, the overall five-year survival rate of patients diagnosed with PC has remained relatively unchanged at between 7% and 9% over the past three decades.

Despite recent improvements in surgery, chemotherapy, and radiation therapy, PDAC, even if it is initially operable, remains a disease with a poor prognosis. This is due to subclinical metastatic disease at onset, early relapse, late stage at onset, a lack of available effective treatment methods, and the lack of a biomarker that can detect this cancer in its early or preinvasive form. Moreover, inflammation of the pancreas is considered a long-term risk factor, and chronic pancreatitis can increase the risk of PDAC by up to 20 times. The poor prognosis is also explained by the complex biology surrounding the extensive desmoplastic microenvironment of a PDAC tumor, which leads to hypo-vascularization, hypoxia, poor drug delivery, and ineffective treatment methods [38]. Currently, there is little few data on the association between the intestinal microbiota and the occurrence of PDAC. Several hypotheses have been put forward that attempt to explain it. This may be because the ducts of the pancreas open and the duodenum and the composition of the microbiota of this part of the intestine is very poorly studied. Moreover, there is evidence of the presence of microbiota directly in the excretory duct of the pancreas, and it is these microbiota that can play a determining role in the development of pathological conditions of the pancreas [39,40,41]. However, several studies have indicated that there are several bacteria genera and species associated with the risk of developing pancreatic cancer, including *Acinetobacter*, *Sphingopyxis*, *Pseudomonas aeruginosa*, and *Fusobacterium* [42,43,44]. Moreover, there is evidence that the microbiota of pancreatic cancer patients was dominated by *Proteobacteria*, *Synergistetes*, and *Euryarchaeota*, which also signifies the role of these phyla in the development of PDAC [45].

## 7. Gut Microbiome in Cancer Immunotherapy

Fecal microbiota transplantation, the short-term dietary manipulation of tryptophan, and oral 3-IAA administration have increased the efficacy of chemotherapy in humanized gnotobiotic mouse models of PDAC, and there was a significant correlation between the level of 3-IAA and the efficacy of therapy in two independent human PDAC cohorts. It has been shown that the efficacy of 3-IAA and chemotherapy is determined by neutrophil-derived myeloperoxidase. Myeloperoxidase oxidizes 3-IAA, which, in combination with chemotherapy, induces the downregulation of the ROS-degrading enzymes glutathione peroxidase 3 and glutathione peroxidase 7. All of this results in the accumulation of ROS and the downregulation of autophagy in cancer cells, which compromises their metabolic fitness and, ultimately, their proliferation. In rare patients with localized PDAC (who tend to be long-term survivors), bacteria can translocate from the intestine into the tumor and control anti-tumor immune activation. However, most patients suffering from aggressive immunotherapy-resistant mPDAC are treated with polychemotherapy, and it is, at present, unclear whether and how the microbiota or dietary habits affect its efficacy. Moreover, despite significant advances in immunotherapy for the treatment of many cancers, the progress of immunotherapy with PDAC has been difficult. It has been shown that microbiota influences the response to immunotherapy, regulates immune checkpoints, and promotes cancer cell escape from the immune system [46].

For example, bacterial suppression has been associated with the increased expression of PD-1 and the increased efficacy of checkpoint-targeted immunotherapy, with a synergistic effect on tumor size and an increase in T-cell activity [47]. In a recent study, it was confirmed that the presence of Megasphaera, which is capable of producing SCFA in tissues, showed a better response to anti-PD-1 therapies [48].

In summary, the microbiota-based approach is an important branch in the treatment of PDAC as it significantly influences the efficacy of chemotherapy and immunotherapy. Microbes are associated with approximately 10–20% of human cancers and are capable of causing carcinogenesis.

The summary data discussed in the article are presented in Table 1.

## 8. Conclusions

Scientific research in recent years has revealed the essential role of the human microbiome in carcinogenesis. The microbiome of the gastrointestinal tract is most actively involved in the pathogenesis of the malignant neoplasms of the digestive system due to changes in the quantitative and qualitative compositions of the microbiota and increases in the production of bacterial metabolites. In the table provided (Table 1), we present a compilation of human gut microbiota species and their respective roles in health, as well as their associations with various pathological conditions, including obesity, diabetes, and cancer. This overview sheds light on the intricate interplay between the gut microbiota and human health, highlighting both protective and pathogenic aspects. Understanding these microbial contributions can pave the way for targeted interventions and therapeutic strategies in the context of these prevalent and complex health conditions. Unfortunately, the microbiological landscape of the intestine as revealed by dozens of studies is very diverse, and a comprehensive analysis of the relationship between certain bacteria and diseases can hardly be interpreted at a deeper physiological level without functional studies or strictly controlled clinical trials that take into account many environmental variables.

The data on the relationship between the composition and metabolic characteristics of the microbiome in various oncological diseases have opened up prospects for their use in the diagnosis, treatment, and prevention of malignant neoplasms and justify the need for further research in this area. To overcome the lack of such data, we are conducting a clinical study on changes in the intestinal microbiota and circulating tumor DNA during the treatment of hormone-negative breast cancer and pancreatic cancer.

The scheme created based on the literature analysis (Figure 1) offers a visual narrative of the intricate web of interactions between the gut microbiota and significant systemic health concerns. It elucidates the multifaceted relationships that underlie these conditions, illustrating how the gut microbiota impacts each of them. In this framework, the gut microbiota could be considered a central player, influencing obesity through its effects on weight regulation, diabetes through its influence on insulin sensitivity, and cancer through its involvement in chronic inflammation. Thus, the gut microbiota governs many essential processes in the development of disease. This serves as a compelling reminder of the vital role the gut microbiota plays in shaping our overall health and the potential for innovative interventions targeting these microbial communities to mitigate disease risk and enhance well-being.

## Figures and Tables

**Table 1:** Microbiological signatures of chronic conditions and oncological diseases of the gastrointestinal tract.

Associated Bacteria	Protective Bacteria	Ref.
Obesity		
*Lactobacillus reuteri*	*Christensenellace*	[26]
	*Akkermansia*	[24]
*Firmicutes*, *Fusobacteria*, *Proteobacteria*, and *Mollicutes*		[25]
Diabetes Meletus Type 2		
*Bacteroides caccae*, *Clostridiumhathewayi*, *Clostridiumramosum*, *Clostridiumsymbiosum*, *Eggerthella lenta*, *Escherichia coli*, *Akkermansia muciniphila*, and *Desulfovibrio* sp.	*Clostridiales* sp. SS3/4, *Eubacterium rectale*, *Faecalibacterium prausnitzii*, *Roseburia intestinalis*, and *Roseburia inulinivorans*	[28]
*Firmicutes*, *Bacteroidetes*, *Proteobacteria*, *Actinobacteria*		[29]
*Ruminococcus*, *Fusobacterium*, and *Blautia*	*Bifidobacterium*, *Bacteroides*, *Faecalibacterium*, *Akkermansia*, and *Roseburia*	[30]
Colorectal Cancer		
*Streptococcus bovis*, enterotoxigenic *Bacteroides fragilis*, *Fusobacterium nucleatum*, *Enterococcus faecalis*, *Escherichia coli*, and *Peptostreptococcus anaerobius*	*Roseburia*	[35]
*Fusobacterium nucleatum*		[34]
Pancreatic Cancer		
*Acinetobacter*, *Pseudomonas*, and *Sphingopyxis*		[40]

## Data Availability

Data sharing is not applicable to this article as no new data were created or analyzed in this study.
